# Adventure Thrills are Addictive

**DOI:** 10.3389/fpsyg.2015.01915

**Published:** 2015-12-16

**Authors:** Ralf C. Buckley

**Affiliations:** International Chair in Ecotourism Research, Griffith School of Environment, Griffith UniversityGold Coast, QLD, Australia

**Keywords:** sports, outdoor, recreation, risk, rush

People can become addicted to behaviors as well as substances (Marks, 1990; Brown, 1997; Elster, 2000; Ranieri, 2011; Berczik et al., 2012; McNamara and McCabe, 2012; Lichtenstein et al., 2014). Behavioral addictions may have social as well as endocrine components, and may include antisocial activities such as crime and gambling, relatively neutral activities such as videogames and internet use, and positive addictions such as sport and exercise (Lichtenstein et al., 2014). Here I argue that participants in many forms of adventure recreation also experience particularly powerful behavioral addictions. This leads them to devote continually increasing proportions of time and resources to carrying out their preferred activities at higher and higher levels of skill, with higher and higher physical risk.

High-risk high-skill adventure recreation has been analyzed previously from the perspectives of commercial tourism products (Buckley, 2007); participant emotions and personalities (Faullant et al., 2011; Pomfret, 2011; Houge Mackenzie and Kerr, 2013), and participant motivations and experiences (Buckley, 2012, 2014a,b, 2015). Behavioral addiction has been diagnosed and examined for addiction to exercise (Berczik et al., 2012; McNamara and McCabe, 2012; Lichtenstein et al., 2014), but not specifically for high-risk adventure activities. Here, therefore, I test the behaviors of adventure participants against medical, mental-health and sports-science criteria for behavioral addictions (Marks, 1990; Brown, 1997; Elster, 2000; Ranieri, 2011; Berczik et al., 2012; McNamara and McCabe, 2012; Lichtenstein et al., 2014).

The criteria used for this test are derived principally from those used by Marks (1990), Brown (1997) and Lichtenstein et al. (2014). According to Marks (1990), the basic symptom of behavioral addiction is a growing psychological pressure for that behavior, which disappears when the addictive behavior is completed, and then returns subsequently. Marks (1990) proposed five testable criteria, namely: increasing preoccupation prior to addictive behavior; strong emotional reward from addictive behavior; withdrawal pains if deprived of addictive behavior; diminishing returns, requiring increasingly extreme addictive behavior; and prioritization of addictive behavior over other obligations and expenditures.

Brown (1997) proposed a similar and approximately corresponding set, namely: salience, conflicts, mood modification, tolerance, withdrawal symptoms, and loss of control. These were the set used most recently by Lichtenstein et al. (2014) in testing for exercise addiction. The two sets differ slightly in degree of specificity and time periods considered. Thus salience refers to the position of an activity in the overall lifestyle of the participant, whereas preoccupation refers to increasing attention immediately prior to undertaking the activity. Prioritization is a general term, whereas conflicts and loss of control refer to specific outcomes from that prioritization. Emotional reward is one specific category of mood modification. In applying these criteria to adventure recreation, both sets are therefore considered jointly, as below.

The adventure activities used for this test are: surfing, sailboarding, kiteboarding, hang-gliding, helicopter snowboarding, and whitewater rafting and kayaking (Buckley, 2007, 2012). At the highest skill levels, these activities also involve high physical risk. There are many other adventure activities, including motorized activities, which are not included in this dataset. Behavioral data are derived from previously reported ethnographic research on extreme, expert, and intermediate level adventure practitioners (Buckley, 2012, 2014a; Buckley and Ollenburg, 2013). This includes a total of >30,000 participant hours of ethnographic observation at intermediate and high skill levels, across multiple adventure activities; and single or repeated interviews with 160 individual adventure exponents at expert and intermediate levels (Buckley, 2012, 2014a; Buckley and Ollenburg, 2013). Data from these sources are summarized below against six criteria for addictive behavior.

The six criteria are considered below in order, from more general social-behavioral to more specific physiological-biochemical. The first group of criteria includes salience, self-image, selective association and discretionary expenditure. Data from numerous individuals and multiple adventure activities, as outlined above, show that adventure enthusiasts identify themselves through their preferred activities, pick their friends and partners from the same activity, and judge themselves on adventure peer esteem. They spend their money and allocate their discretionary time largely on adventure equipment and trips, including commercial tourism products, to practice their preferred activities. They organize their lives to minimize other costs, to save money for these activities. Their lives revolve around these activities, and they spend considerable time planning, selecting and designing them.

The second group of criteria is preoccupation with the activity prior to participation, and prioritization of the preferred activity over other obligations. Both of these are also very widespread amongst proponents of adventure activities. Many ski slopes and surf beaches, for example, are watchable continuously through live webcams; and wave heights, wind speeds and river flows are updated continually from automatic recorders. Surfers, skiers, snowboarders, sailboarders, kiteboarders and kayakers watch these feeds and cameras routinely, continually, and indeed compulsively, during work or social activities, and cancel or abandon everything else when conditions look favorable.

In coastal surf towns, surfers don't show up for work when the surf is good, even if they put their jobs at risk. In ski towns and resorts, new-fallen snow overrides all social obligations. At some iconic sailboarding and kiteboarding sites, in order to retain skilled staff, businesses offer employment contracts containing wind clauses, which allow sailboarders and kiteboarders to leave work when wind speed exceeds a fixed threshold. At sites where hangglider pilots must predict wind conditions in advance in order to arrive in time for a relatively short favorable window, they will routinely abandon social or professional events at a moment's notice when such conditions are predicted. Individual adventure practitioners commonly depart mid-work or mid-meal when conditions become suitable, and have been known to abandon social obligations even including a wedding still in progress.

The third criterion is mood modification and emotional reward. Both of these occur powerfully amongst skilled adventure practitioners. In adventure tourism destinations, many inhabitants show major mood swings depending on recent conditions. If they have been able to surf, ski, ride or paddle, they are happy. During adventure activities, air-punch “claim” gestures and uninhibited yells of triumph and excitement are commonplace, even from otherwise undemonstrative individuals. Practitioners who have just engaged successfully in high-thrill adventure activities become emotionally immunized, for periods of hours or even days, against negative emotions which would otherwise arise from otherwise depressing news. Examples including loss of employment and termination of relationships. All these effects indicate powerful biochemical as well as behavioral and psychological responses, demonstrating addiction at a fundamental level.

The fourth criterion is tolerance, or diminishing rewards, from continuing exposure to an addictive behavior. This is indeed commonplace amongst adventure practitioners.

Individuals learning adventure activities initially experience thrill from relatively low-key achievements. As their skills and experience increase, however, they need more and more difficult challenges to achieve the same emotional response. This may include, for example, larger and steeper waves, rapids or snow slopes; stronger winds; more difficult terrain, and so on (Buckley, 2012, 2014a). This is a key reason why they improve skills to as to overcome higher risks (Buckley, 2012). The psychological significance of this is shown by the behavior of expert adventure tourists, who pay heavily for adventure tourism operators to take them to uncrowded sites with world-class conditions. Some adventure participants invest several hours every day over many years to achieve relevant skills, so that they may then be rewarded by very brief extreme experiences (Buckley, 2014b): a single moment paid for by a lifetime of investment.

The fifth criterion for recognizing addictive behavior is withdrawal symptoms, when the activity is not available. Such symptoms are widespread and powerful. When adventure practitioners have been unable to enjoy their preferred activities, e.g., through work or weather conditions, they become depressed and bad-tempered. When high winds or heavy snowfalls keep heli-skiers grounded, for example, they suffer great gloom. When conditions improve, gloom is replaced by euphoria and higher tolerance to risk, e.g., from avalanches associated with new heavy snowfalls. At crowded surf breaks, some surfers succumb to “surf rage” through frustration and fury if others steal their waves. When adventure practitioners are unexpectedly prevented from practicing their preferred activities because of broken, mislaid or forgotten equipment, they suffer powerful distress, with physiological symptoms such as pain, perspiration and palpitations. All of these are strong indicators of addiction.

At intermediate and expert level, therefore, skilled participants in high-risk adventure recreation activities do indeed show symptoms of strong behavioral addiction. Adventure aficionados become addicted to thrills, at least as strongly as participants can become addicted to sports or exercise (Berczik et al., 2012; McNamara and McCabe, 2012; Lichtenstein et al., 2014).

From a marketing perspective, adventure enterprises catering for experienced clients are thus essentially selling fixes to addicts, a strategy which depends only on the cash reserves of the addicts. Experienced clients, however, form only a small component of the adventure tourism sector overall (Buckley, 2007; Faullant et al., 2011; Pomfret, 2011). For less experienced clients, who are far more numerous (Buckley, 2007), motivations are largely social (Buckley, 2012). Most commercial adventure enterprises rely principally on inexperienced clients to generate cash flow (Buckley, 2007). Those that offer high-skill low-volume activities to wealthy clients, however, may indeed rely on addiction as part of their marketing mix. Heli-skiing is perhaps a prime example.

From a psychological research perspective, many adventure aficionados do indeed behave as addicts. Addiction to the thrills obtained through adventure recreation can therefore provide a large pool of potential subjects for psychological, neurophysiological and biochemical research on behavioral addictions, with straightforward ethical considerations in research design. This offers addiction researchers new opportunities for controlled experimental designs involving direct measurements on well-informed and willing volunteers.

## Conflict of interest statement

The author declares that the research was conducted in the absence of any commercial or financial relationships that could be construed as a potential conflict of interest.
